# Development of Neutral pH-Responsive Microgels by Tuning Cross-Linking Conditions

**DOI:** 10.3390/s20123367

**Published:** 2020-06-14

**Authors:** Satoshi Okada, Satoko Takayasu, Shunsuke Tomita, Yoshio Suzuki, Shinya Yamamoto

**Affiliations:** 1 Laboratory for Chemistry and Life Science Institute of Innovative Research, Tokyo Institute of Technology, 4259 Nagatsuta-cho, Midori-ku, Yokohama, Kanagawa 226-8503, Japan; 2 JST, PRESTO, 4259 Nagatsuta-cho, Midori-ku, Yokohama, Kanagawa 226-8503, Japan; 3 Health and Medical Research Institute, National Institute of Advanced Industrial Science and Technology (AIST), 1-1-1 Higashi, Tsukuba, Ibaraki 305-8566, Japan; s-takayasu@aist.go.jp (S.T.); s.tomita@aist.go.jp (S.T.); suzuki-yoshio@aist.go.jp (Y.S.); 4 Human Informatics and Interaction Research Institute, National Institute of Advanced Industrial Science and Technology (AIST), 1-1-1 Umezono, Tsukuba, Ibaraki 305-8568, Japan; yamamoto-s@aist.go.jp

**Keywords:** pH-responsive microgels, NMR relaxation time, volume phase transition, MRI

## Abstract

Polymer microgels that respond in a range of neutral pH can be useful for the development of molecular imaging tools and drug-delivery carriers. Here, we describe a simple approach in developing microgels that undergo volume phase transitions and substantial nuclear magnetic resonance (NMR) relaxometric changes within a narrow pH range of 6.4 to 7.4. The pH-responsive microgels were synthesized using methacrylic acid and a series of ethylene glycol dimethacrylate cross-linkers with repeating units of ethylene glycol that range from one to four. NMR relaxometry demonstrated that the transverse relaxation time (*T*_2_) of a suspension containing microgels that were cross-linked with diethylene glycol dimethacrylate sharply decreases at the pH where volume phase transition occurs. The polymer microgels cross-linked with 40 and 45 mol% of diethylene glycol dimethacrylate caused about 50% *T*_2_ reduction with decreasing pH from 6.8 to 6.4. These results demonstrated that responses of microgels to a range of neutral pH can be easily tuned by using appropriate cross-linkers with certain cross-linking degree. This approach can be useful in developing highly sensitive molecular sensors for magnetic resonance imaging (MRI) of tissue pH values.

## 1. Introduction

Magnetic resonance spectroscopy has revealed disorders of acid-base balance in pathologic tissues, although its spatial and temporal resolution is inferior to conventional magnetic resonance imaging (MRI) [1,2,3]. MRI contrast agent-based pH sensors can be powerful tools for highly sensitive pH imaging [4,5,6,7,8,9,10]; however, it is still difficult to make contrast agents respond within a narrow range of physiological pH. The detectability of the pH difference between 6.4 and 7.4 is important for the identification of diseases such as cancer, kidney disease, and cardiac ischemia because these pH values are consistent with the pH values observed in pathological and normal tissues, respectively [11,12]. To develop neutral pH-responsive MRI sensors, synthetic cross-linked polymers were considered as promising materials because their volume phase transitions cause significant changes in the transverse relaxation time (*T*_2_) of water [13,14,15]. The shrunken state of cross-linked polymer microgels enhances *T*_2_ relaxation of solvent water protons more efficiently than its swollen state. Precipitation polymerization is a simple way to synthesize monodisperse microgels, although there are few reports about neutral pH-responsive polymers synthesized by this method [16,17,18,19,20,21]. Here, we present a simple approach in developing microgels that undergo volume phase transition in a narrow range of physiological pH and their application to neutral pH sensors for nuclear magnetic resonance (NMR)/MRI.

## 2. Materials and Methods

### 2.1. Reagents

Methacrylic acid (MAA), *N*,*N’*-methylenebisacrylamide (MBAA), ethylene glycol dimethacrylate (EGDMA), 2,2′-azobisisobutyronitrile (AIBN), and acetonitrile were obtained from FUJIFILM Wako Pure Chemical Co. (Osaka, Japan). Diethylene glycol dimethacrylate (DEGDMA), triethylene glycol dimethacrylate (TEGDMA), and tetraethylene glycol dimethacrylate (TETEGDMA) were obtained from Tokyo Chemical Industry Co., Ltd. (Tokyo, Japan). All of the reagents were of the best grade available. The cross-linkers were purified with active basic aluminum oxide 60 (Sigma-Aldrich Co. LLC., St. Louis, MO, USA) to remove polymerization inhibitors.

### 2.2. Synthesis

The cross-linked polymer microgels were synthesized by precipitation polymerization [19,20]. In a typical synthesis of 20 mol% cross-linked polymers, MAA (490 mg, 5.69 mmol), a cross-linker (1.42 mmol), and AIBN initiator (13 mg, 0.08 mmol, 1 mol% of total monomers) were dissolved in 80 mL of acetonitrile. The mixture was refluxed under nitrogen for 1 h. Then, the mixture was centrifuged at 20,000× *g* and washed by acetonitrile three times. The white precipitate was dried in vacuo to obtain 100–200 mg of polymers. To synthesize other polymers with different cross-linking degree, the molar ratio of the cross-linker to MAA was changed accordingly; the total mole of the cross-linker and MAA was kept at 7.12 mmol in 80 mL of acetonitrile. The stoichiometric ratio of the cross-linker to the total monomers was almost consistent with the cross-linking degree calculated from elemental analysis, except for the MBAA-based polymer, where the cross-linking degree was 30 mol%.

### 2.3. Nuclear Magnetic Resonance (NMR) Relaxometry

Longitudinal relaxation time (*T*_1_) and transverse relaxation time (*T*_2_) were measured using a Spinsolve ULTRA 43 MHz ^1^H-NMR (Magritek Ltd., Wellington, New Zealand) in 500 μL of 100 mM phosphate buffer (see Supplementary Text about NMR relaxation). The inversion-recovery (IR) and Carr–Purcell–Meiboom–Gill (CPMG) pulse sequences were used to measure *T*_1_ and *T*_2_, respectively. The parameters in the inversion recovery (IR) pulse sequence were as follows: number of scans = 2, acquisition time = 1.6 s, repetition time = 7 s, maximum inversion time = 5 s, number of steps = 21. The parameters in the CPMG sequence pulse sequence were as follows: number of scans = 4, acquisition time = 0.8 s, repetition time = 4 s, CPMG echo time = 1 ms, final echo time = 2 s, number of steps = 20. 

### 2.4. Magnetic Resonance Imaging (MRI)

MRI was performed using MRmini SA1506 scanner (DS Pharma Biomedical Co., Ltd., Osaka, Japan) equipped with a 1.5 T permanent magnet and a radiofrequency (RF) coil of 38.5 mm inner diameter. A multislice spin echo pulse sequence was used to obtain *T*_2_-weighted images with parameters including the number of averages = 1, matrix size = 128 × 256, field of view = 2 cm × 4 cm, slice thickness = 2 mm, repetition time (TR) = 2000 ms, and echo time (TE) = 150 ms.

### 2.5. Transmission Electron Microscopy (TEM)

A 5 μL Milli-Q water containing 0.01 wt% cross-linked polymer was dropped on a formvar-coated grid stabilized with evaporated carbon film (STEM Co., Ltd., Tokyo, Japan) and then dried under vacuum. TEM images were captured using JEM-2100F (JEOL Ltd., Tokyo, Japan) operated at 200 kV.

## 3. Results and Discussion

### 3.1. Synthesis of Cross-Linked Polymer Microgels

**Scheme 1:**
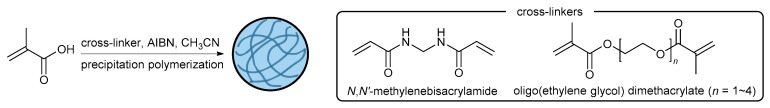
Synthesis of the pH-responsive microgels.

We expected that the phase transition behavior of microgels would be tuned by changing the molar ratio of a cross-linker to an ionic monomer as well as the type of cross-linker. On the basis of this premise, we synthesized poly(methacrylic acid) cross-linked with a series of ethylene glycol dimethacrylate with repeating units ranging from one to four by precipitation polymerization (Scheme 1).

The synthesized polymer microgels were divided into two groups based on the type of cross-linker used and the degree of cross-linking (Table 1). The microgels in Group 1 were synthesized using five different types of cross-linkers: *N*,*N’*-methylenebisacrylamide (MBAA), ethylene glycol dimethacrylate (EGDMA), diethylene glycol dimethacrylate (DEGDMA), triethylene glycol dimethacrylate (TEGDMA), and tetraethylene glycol dimethacrylate (TETEGDMA). In Group 1, the molar ratio of the cross-linkers to the total monomers was fixed at 20 mol% in reaction stoichiometry, except for the EGDMA-based microgel as it produced low yield at the same mol%. The polymer cross-linked by MBAA showed a slightly higher degree of cross-linking than its stoichiometric ratio, whereas the cross-linking degree of the other microgels was almost consistent with their corresponding ratios. The microgels in Group 2 were synthesized using the DEGDMA cross-linker only but with different cross-linking degrees of 20, 30, 40, and 45 mol%. The 20 mol% DEGDMA-based microgel belongs to both groups. We tried to synthesize the microgels cross-linked by 30 mol% of TEGDMA and TETEGDMA but they formed a huge aggregate during reaction. The reaction mixture using longer cross-linkers and larger amounts of cross-linkers became more turbid. This indicates the formation of highly polymerized compounds due to the high reactivity of the cross-linkers [22].

### 3.2. TEM Images and Volume Phase Transition Behaviors

**Figure 1:**
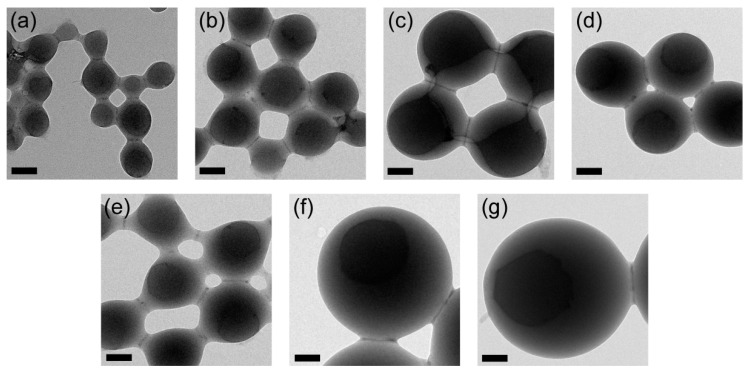
Transmission electron microscopy (TEM) images of poly(methacrylic acid) cross-linked with (**a**) 40 mol% EGDMA, (**b**) 20 mol% DEGDMA, (**c**) 20 mol% TEGDMA, (**d**) 20 mol% TETEGDMA, (**e**) 30 mol% DEGDMA, (**f**) 40 mol% DEGDMA, and (**g**) 45 mol% DEGDMA. Scale bars = 100 nm.

TEM showed that the cross-linked polymers in both groups have a uniform and spherical structure (Figure 1). We were unable to obtain clear images of the MBAA-based microgel due to difficulties in focusing. The mean diameters of Group 1 microgel particles increased from 120 nm up to 330 nm as the length of the cross-linkers increased (Figure 1a–d and Table S1). The microgels with longer cross-linkers also showed a clearer outline. The TEM images of Group 2 microgel particles show that a higher degree of cross-linking results in a larger particle size from 160 nm to 580 nm and a more distinct structure (Figure 1b,e–g and Table S1). Interestingly, a core-shell structure was observed in all the polymers. This might be due to an uneven distribution of cross-linking density caused by the reactivity difference between MAA and the cross-linkers [22,23].

**Figure 2:**
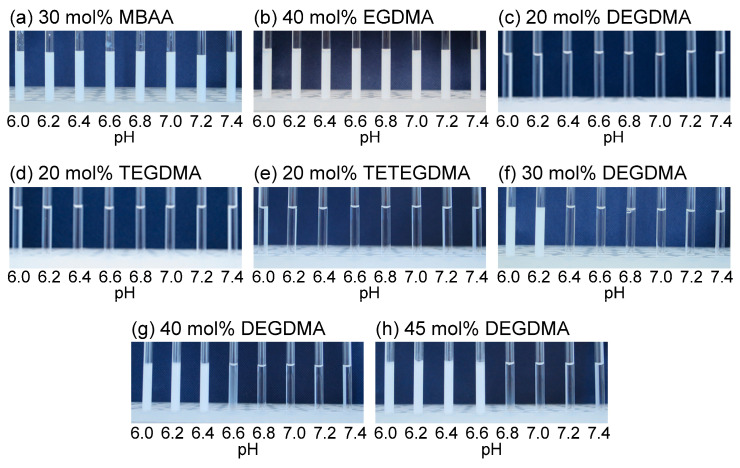
The phase transition behaviors of the poly(methacrylic acid) cross-linked with (**a**) 30 mol% MBAA, (**b**) 40 mol% EGDMA, (**c**) 20 mol% DEGDMA, (**d**) 20 mol% TEGDMA, (**e**) 20 mol% TETEGDMA, (**f**) 30 mol% DEGDMA, (**g**) 40 mol% DEGDMA, and (**h**) 45 mol% DEGDMA in 100 mM phosphate buffer, pH 6.0 to 7.4 with 0.2 increments at room temperature. [microgel] = 0.50 wt%.

A volume phase transition behavior was confirmed by testing suspensions with 0.50 wt% microgel concentration against 100 mM phosphate buffer with pH ranging from 6.0 to 7.4 at 0.2 increments (Figure 2). In Group 1, the MBAA- and EGDMA-based microgel suspensions were turbid throughout the pH range (Figure 2a,b), whereas the other microgel suspensions were transparent (Figure 2c–e). In contrast, a phase transition within the pH range of 6.2 to 6.6, with 0.2 pH differences, was clearly visible in Group 2 (Figure 2f–h). The transition pH of the 30, 40, and 45 mol% DEGDMA-based microgels were 6.2, 6.6, and 6.6, respectively; however, the 40 mol% DEGDMA-based microgel showed an intermediate state at pH 6.6 (Figure 2g).

**Figure 3:**
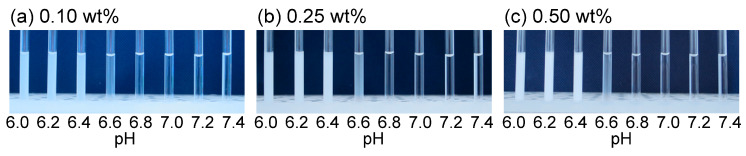
Phase transition behaviors of (**a**) 0.10 wt%, (**b**) 0.25 wt%, and (**c**) 0.50 wt% of the 40 mol% DEGDMA-based microgel in 100 mM phosphate buffer, pH 6.0 to 7.4 with 0.2 increments at room temperature.

Figure 3 shows the volume phase transition behaviors of the 40 mol% DEGDMA-based microgel suspensions in different concentrations (0.10 to 0.50 wt%). The suspensions became less turbid with decreasing concentration; however, the transition at pH 6.6 did not change, indicating that the microgel function could be kept relatively stable even if they were diluted after injection into the body.

### 3.3. NMR Relaxometric Properties at Neutral pH

**Figure 4:**
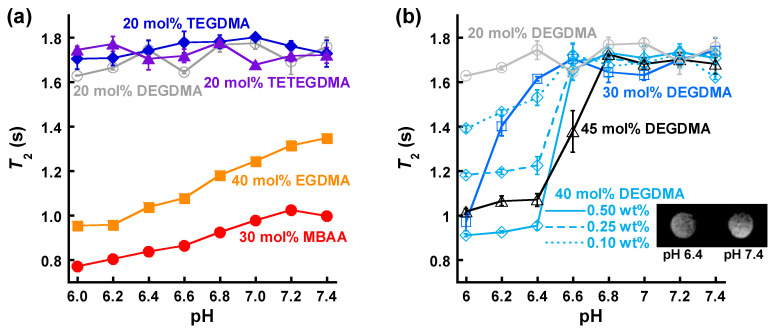
Transverse relaxation time (*T*_2_) of microgel suspensions of (**a**) Group 1 and (**b**) Group 2 in the pH range of 6.0–7.4 at 28 °C (mean ± standard error of the mean (SEM), *n* = 3). The microgel cross-linked with 20 mol% DEGDMA is shown in both figures. The inset in (**b**) is a *T*_2_-weighted image of the 40 mol% DEGDMA-based microgel in 100 mM phosphate buffer (pH 6.4 and 7.4) at room temperature. [microgel] = 0.50 wt% unless otherwise noted.

The transverse relaxation time (*T*_2_) of 0.50 wt% microgel suspensions was measured using a 43 MHz ^1^H-NMR. In Group 1, *T*_2_ was gradually reduced in the MBAA- and EGDMA-based microgels with decreasing pH (Figure 4a). The reductions in *T*_2_ reached up to 0.3 s as the pH was adjusted from 7.4 to 6.0. Other polymers in Group 1 did not produce a significant reduction in *T*_2_. On the other hand, Group 2 microgels showed sharp reductions in *T*_2_ at pH 6.6–6.8, except for the 20 mol% DEGDMA-based microgel (Figure 4b). The *T*_2_ reductions caused by 40 mol% and 45 mol% DEGDMA-based microgels were 0.8 and 0.7 s, respectively, in the narrow pH range of 6.4 to 6.8. The threshold pH of *T*_2_ jump shifted from 6.2 to 6.6 with increasing cross-linking degree and correlated with the volume phase-transition behaviors (Figure 2f–h). These results demonstrated that the threshold pH can be adjusted by tuning the cross-linking degree and using an appropriate cross-linker according to the pH of the target region. A *T*_2_-weighted MRI using a 1.5 T scanner showed that the microgel cross-linked with 40 mol% DEGDMA produced significant changes in the MRI signal from pH 6.4 to pH 7.4 (Figure 4b inset); this indicates that a 40 mol% DEGDMA-based microgel can be a useful MRI sensor in detecting slight pH difference in pathologic tissue and normal tissue [12].

The phase transition depends on electrostatic repulsion of deprotonated carboxy groups and intramolecular attractive force such as van der Waals interactions of the side chains [24]. The higher degree of cross-linking can increase the intramolecular attractive force. As a result the phase transition pH shifted to a higher value than apparent p*K*_a_ of non-cross-linked poly(methacrylic acid), which is around 6 [24]. The microgels cross-linked by 40 mol% and 45 mol% DEGDMA could have well-balance between hydrophilicity of the ethylene glycol moieties and hydrophobicity of the methyl groups to respond in the neutral pH. On the other hand, the other cross-linkers were unlikely to be utilized to achieve this balance. TEGDMA and TETEGDMA formed a huge aggregate if their reaction stoichiometry was more than 30 mol% because of their high reactivity. EGDMA and MBAA may be too short and less flexible to swell beyond the intramolecular attractive force.

**Figure 5:**
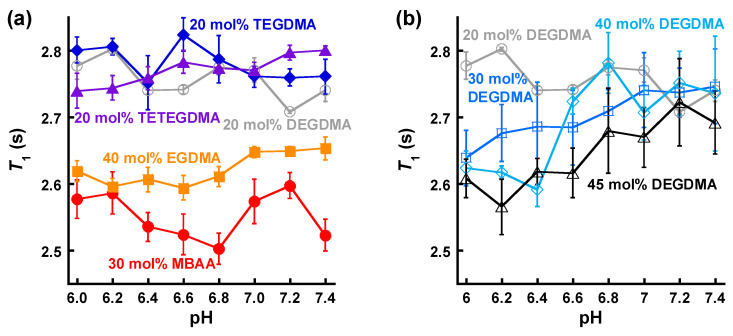
Longitudinal relaxation time (*T*_1_) of microgel suspensions of (**a**) Group 1 and (**b**) Group 2 in the pH range of 6.0–7.4 at 28 °C (mean ± SEM, *n* = 3). The microgel cross-linked with 20 mol% DEGDMA is shown in both figures. [microgel] = 0.50 wt%.

The concentration dependence of *T*_2_ was tested using 0.10 and 0.25 wt% suspensions of the 40 mol% DEGDMA-based microgel (Figure 4b). The 0.25 wt% suspension showed a *T*_2_ jump between pH 6.4 and 6.6, which was similar to that of 0.50 wt% suspension. In contrast, the 0.10 wt% suspension showed moderate *T*_2_ changes from pH 6.0 to 6.6, although the phase transition pH was maintained at approximately 6.6 (Figure 3a). Therefore, *T*_2_ reductions may be caused not only by volume phase transition but also by other pH-dependent effects such as proton chemical exchange via functional side groups [25,26,27]. Group 1 and 2 microgel suspensions showed little changes in longitudinal relaxation time (*T*_1_) compared with the polymer-free buffer (*T*_1_ ≈ 2.8, data not shown) (Figure 5). This could be due to the extremely slow motion of bound waters on the microgel surface [28,29,30,31].

## 4. Conclusions

We demonstrated the development of the polymer microgels that undergo volume phase transition in the narrow range of neutral pH by tuning cross-linking conditions in precipitation polymerization. The microgels based on the appropriate cross-linkers and cross-linking degree caused substantial *T*_2_ changes around the transition pH. *T*_2_-weighted MRI showed that the 40 mol% DEGDMA-based microgel permits detection of a weakly acidic condition. This approach might be useful for producing a versatile platform, especially for the development of pH-responsive MRI sensors. For future application, a conjugation with paramagnetic metal chelates and polymer encapsulation of magnetic nanoparticles would be potentially important in enhancing the sensitivity of sensors [32,33,34,35,36].

## Figures and Tables

**Table 1:** The groups of poly(methacrylic acid) based on different types of cross-linkers and different degrees of cross-linking.

Group	Cross-Linker and Repeating Unit (*n*)	Cross-Linking Degree (mol%)
1	MBAA	30
1	EGDMA (*n* = 1)	40
1, 2	DEGDMA (*n* = 2)	20
1	TEGDMA (*n* = 3)	20
1	TETEGDMA (*n* = 4)	20
2	DEGDMA (*n* = 2)	30
2	DEGDMA (*n* = 2)	40
2	DEGDMA (*n* = 2)	45

## Supplementary Materials

The following are available online at https://www.mdpi.com/1424-8220/20/12/3367/s1: Supplementary Text: Longitudinal relaxation time (*T*_1_) and transverse relaxation time (*T*_2_)., Table S1: Mean diameters of the microgels calculated from TEM images.
