# Supplementary material for: Development of Neutral pH-Responsive Microgels by Tuning Cross-Linking Conditions
Source: Sensors (Basel). 2020 Jun 14;20(12):3367. doi: 10.3390/s20123367 (PMC7349689; doi:10.3390/s20123367)
Supplement: Supplementary file 1 [file sensors-20-03367-s001.pdf]

Supplementary Materials

Development of Neutral pH-Responsive Microgels by Tuning Cross-Linking Conditions

Satoshi Okada*, Satoko Takayasu, Shunsuke Tomita, Yoshio Suzuki and Shinya Yamamoto

*e-mail: sokada@res.titech.ac.jp

Contents:

Supplementary Text: Longitudinal relaxation time (T_1) and transverse relaxation time (T_2).
Supplementary Table S1: Mean diameters of the microgels calculated from TEM images.

Longitudinal relaxation time (T_1) and transverse relaxation time (T_2).

^{1}H-MRI signal intensity depends on two relaxation processes of the net magnetization (M_0) of water proton spins. M_0 is initially parallel with static magnetic field (Z-direction) but there is no net magnetization on XY plane. By applying radio frequency (RF) pulse, proton spins are excited to a higher energy state involved with phase coherence and M_0 is tilted out from the Z-direction. At the end of RF pulse, the excited proton spins gradually return to thermal equilibrium state and the net magnetization is also relaxed to the initial state M_0. This relaxation process is separated into two components, Z-direction and XY-direction. The net magnetization recovery of the Z-direction is termed longitudinal relaxation defined as $M_z = M_0[1\text{-exp}(-t/T_1)]$; T_1 is the time required for M_z to approach 63% of M_0. Longitudinal relaxation is energy exchange between proton spins and surrounding lattice. On the other hand, the net magnetization decay on the XY-plane is termed transverse relaxation. This process is a loss of phase coherence and defined as $M_{xy} = M_0\text{exp}(-t/T_2)$; T_2 is the time required for M_{xy} to approach 37% of M_0. General MRI contrast agents significantly reduce T_1 and T_2 due to a dipole-dipole interaction between their electron spins and proton nuclear spins and/or distortion of local magnetic field.

Table S1. Mean diameters of the microgels calculated from TEM images.

Group	Microgels cross-linked by	Mean diameter ± SEM
1	40mol% EGDMA	120 ± 4 nm (n = 69)
1, 2	20mol% DEGDMA	161 ± 2 nm (n = 76)
1	20mol% TEGDMA	293 ± 4 nm (n = 31)
1	20mol% TETEGDMA	326 ± 13 nm (n = 54)
2	30mol% DEGDMA	227 ± 5 nm (n = 44)
2	40mol% DEGDMA	564 ± 16 nm (n = 35)
2	45mol% DEGDMA	579 ± 15 nm (n = 14)
